# Detection of infectious agents in lungs of slaughtered pigs in association with cranioventral pulmonary consolidation

**DOI:** 10.2478/jvetres-2025-0049

**Published:** 2025-09-22

**Authors:** Arkadiusz Dors, Małgorzata D. Klimowicz-Bodys, Zbigniew Kuberka, Agnieszka Nowak, Sylwia Zębek, Kinga Urbaniak, Katarzyna Szymanek, Anna Rząsa

**Affiliations:** 1 Department of Preclinical Sciences and Infectious Diseases, Faculty of Veterinary Medicine and Animal Sciences, Poznań University of Life Sciences, 60-637 Poznań, Poland; 2 Division of Infectious Diseases of Animals and Veterinary Administration, Department of Epizootiology and Clinic of Birds and Exotic Animals, Faculty of Veterinary Medicine, Wrocław University of Environmental and Life Sciences, 50-366 Wrocław, Poland; 3 Private Practice „BONA – WET”, 63-330 Dobrzyca, Poland; 4 Department of Bacteriology and Bacterial Animal Diseases,; 5 Department of Virology and Viral Animal Diseases, National Veterinary Research Institute, 24-100 Puławy, Poland; 6 Department of Immunology, Pathophysiology and Veterinary Preventive Medicine, Wrocław University of Environmental and Life Sciences, 50-375 Wrocław, Poland

**Keywords:** pigs, lung lesions, cranioventral pulmonary consolidation, *Mycoplasma hyopneumoniae*, PRDC

## Abstract

**Introduction:**

Respiratory diseases have a substantial impact on swine production worldwide. Understanding the relationship between gross lung lesions and the presence of infectious agents is crucial for developing effective disease control strategies that target both primary and secondary pathogens.

**Material and Methods:**

A cross-sectional study was conducted on 22 pig farms in western Poland. Cranioventral pulmonary consolidation (CVPC) in slaughtered pigs was assessed, and 20 lung tissue samples were collected from each herd. The presence of common bacterial and viral respiratory pathogens was identified using PCR-based methods.

**Results:**

The disorder was observed in 79.3% (95% confidence interval 75.3–82.8) of slaughtered pigs across all examined herds. The most frequently detected pathogens at both the herd and individual animal levels were *Glaesserella parasuis, Mycoplasma hyopneumoniae* and porcine circovirus 2. Co-infections involving two or more respiratory pathogens were prevalent, occurring in 100% of herds and 87.7% of individual pigs. Mean CVPC scores were significantly higher in pigs infected with *Mycoplasma hyopneumoniae, Mycoplasma hyorhinis* and porcine reproductive and respiratory syndrome virus 1.

**Conclusion:**

These findings highlight the multifactorial nature of respiratory infections in pigs. Effective control measures should consider the high prevalence of co-infections and their impact on lung lesion severity to improve overall herd health and productivity.

## Introduction

Respiratory diseases significantly impact swine production worldwide, contributing to economic losses due to reduced growth performance, increased treatment costs and higher mortality rates (22, 23). The aetiology of these diseases is often multifactorial, involving complex interactions between bacterial and viral pathogens, environmental conditions and host immunity (28, 37). One of the most prevalent respiratory syndromes affecting pigs is porcine respiratory disease complex (PRDC), characterised by polymicrobial infections leading to chronic lung lesions and decreased growth (31, 35).

Abattoir evaluation of lung lesions is essential for assessing respiratory disease prevalence and severity in swine herds. It aids in detecting subclinical conditions, evaluating risk factors and assessing vaccine efficacy (4, 9, 15). The methods applied to abattoir porcine lung samples are non-invasive and cost-effective, and enable the evaluation of a representative sample of animals within a relatively short time frame (21, 25, 34). Cranioventral pulmonary consolidation (CVPC) is among the most common gross lung lesions observed in slaughtered pigs, and is often associated with infections caused by bacterial and viral agents (10, 25). The presence and severity of CVPC lesions can serve as indicators of herd health status, and signal both past and ongoing respiratory infections (21). Lung lesion scoring at slaughter also proves valuable in the evaluation of the effectiveness of respiratory disease control programmes, as well as productivity and economic efficiency in commercial swine herds, which explains its frequent implementation (30, 33). However, establishing a clear association between the activity of individual pathogens or their combinations and the prevalence and severity of lung lesions remains challenging. Research has been conducted, and several publications are available on this topic (2, 5, 12, 18, 35). However, because of the diversity of pathogens causing PRDC, knowledge in this area remains insufficient.

The objectives of this study were to evaluate the prevalence of CVPC lesions in slaughtered pigs from commercial farms in western Poland and to investigate the association between these lesions and the presence of selected respiratory pathogens. Molecular diagnostic techniques were used to identify key bacterial and viral agents involved in lung pathology, and the identifications made enhance the understanding of the epidemiology of respiratory infections in swine and support the development of effective control measures.

## Material and Methods

### Farms and study design

A cross-sectional study was conducted on pig farms in the western part of Poland (Wielkopolskie voivodeship), in a region with a high density of pig production. This voivodeship was chosen because it is one of the regions with the most intensive pig rearing in the country, and respiratory disorders occur in its swine herds regularly. This study was part of a larger project on swine respiratory diseases (15). The farms were included on a non-random, convenience basis from the veterinary practitioner client database (n = 50 clients) of one of the co-authors. All herd owners were contacted by telephone or through personal communication and invited to participate in the study. Previous occurrence of respiratory diseases or other health issues in the herds was not considered a criterion for herd selection in the study. Those who self-selected and met the criteria of selling at least 75 fatteners in a single batch and not administering antibiotics for at least 14 d prior to slaughter had their farm’s herd enrolled. In total, 22 farrow-to-finish pig herds were involved in the study. These farms varied in size, production type, genetic lines/breeds of pigs kept, biosecurity level, herd health status and preventive programmes implemented. A detailed characterisation of the herds included in the study was presented in a separate publication (15). In each herd, 20 randomly selected fatteners were tagged on an ear. After slaughter, each tagged animal’s lungs were scored in respect of lesions. Simultaneously, samples of these animals’ lung tissue were collected for laboratory testing for the presence of selected swine respiratory pathogens.

### Lung lesion scoring

The assessment of lung lesions in pigs was conducted at the abattoir immediately after the animals were slaughtered. The scoring was always performed by the same person, a trained and experienced veterinarian. The lungs were evaluated for CVPC lesions using the two-dimensional method described by Madec and Kobisch (20). Each lung lobe was visually assessed for the extent of consolidation, and a score from 0 to 4 was assigned accordingly. A score of 0 indicated no visible lesions; 1 was given when less than 25% of the lobe was affected (small focal consolidation); 2 corresponded to 25–49% of the lobe being involved (moderate consolidation); 3 was assigned for 50–74% involvement (extensive consolidation); and 4 reflected nearly complete or complete consolidation, with ≥75% of the lobe affected. The final lung score was the sum of scores from seven lobes, and ranged from 0 to 28. In each herd, lungs from 20 fatteners were scored.

### Sampling

Tissue samples for the study were collected from pigs post slaughter, following a visual assessment of lung lesions. Three cube-shaped lung tissue samples, each with side length of approximately 1.5 cm, were taken from three different lobes: the cranial, middle and caudal. If macroscopic lesions were detected in a given lobe, the sample included the affected tissue along with a small margin of healthy tissue. The lung samples were placed in sterile 50 mL Falcon tubes, labelled and transported under chilled conditions to the laboratory, where they were stored at −70°C until further analysis.

As samples were collected from pigs after commercial slaughter, permission from the Local Ethical Commission for Investigations on Animals was not required for the study described in this manuscript. The sampling in slaughterhouses was carried out with the consent of the local veterinary authorities. Informed consent was obtained from the owners of the farms for samples from their animals to be used in the study.

### Pathogen detection

Lung tissue samples weighing 1–2 g were macerated using an X120 homogenizer (Ingenieurbüro CAT, M. Zipperer, Ballrechten-Dottingen, Germany) in a 50% mass-volume solution, and then 100 μL of the supernatant was collected. Total RNA/DNA was extracted from 100 μL of the supernatant obtained during the homogenisation of tissue samples using the MagNaPure LC automatic extractor and the MagNaPure LC Total Nucleic Acid Isolation Kit (both Roche, Basel, Switzerland) or Genomic Mini Kit (A&A Biotechnology, Gdańsk, Poland) according to the manufacturers’ instructions. The resulting purified filtrate was subjected to PCRs or stored at −70°C until further analysis.

The genetic material of *Actinobacillus pleuropneumoniae* (APP), *Glaesserella parasuis* (GLAS), *Mycoplasma hyopneumoniae* (MHP), *Mycoplasma hyorhinis* (MHR), porcine circovirus 2 (PCV2) and porcine circovirus 3 (PCV3) was detected using individual PCR tests with the Master Mix QuantiTect Probe PCR Kit (Qiagen, Germantown, MD, USA). A list of oligonucleotides used in the reaction and the genes detected for each pathogen is provided in Table 1. The genetic material of *Streptococcus suis* (STREP) was detected using 2× AccuStart II GelTrack PCR SuperMix (Quantabio, Beverly, MA, USA) with the primers noted in Table 1. The PCR products were separated by electrophoresis in 2% agarose gel, and fluorescence gel images were captured under UV light using an EC3 Chemi HR 410 Imaging System (UVP, Upland, CA, USA). In the real-time PCR assay for porcine reproductive and respiratory syndrome virus 1 and 2 (PRRSV1 and PRRSV2), the commercial EZPRRSV MPX 4.0 assay kit (Tetracore, Rockville, MD, USA) was used. For swine influenza A virus (swIAV), a method with separately synthesised primers and a nucleolytic probe was used, along with the QuantiTect Probe RT-PCR Kit (Qiagen, Hilden, Germany). The sequences of the primers used in the reverse transcription PCR reaction for swIAV are shown in Table 1.

**Table 1.** Oligonucleotide primer and probe sequences used for detection of pathogens causing cranioventral pulmonary consolidation in slaughtered Polish pigs

Pathogen	Target (gene)	Oligonucleotide sequence (5′–3′)	Reference
APP	*apxIVA*	apxIVANEST1-F: GGGGACGTAACTCGGTGATT apxIVANEST1-R: GCTCACCAACGTTTGCTCAT apxIVAPr (probe): FAM-CGGTGCGGACACCTATATCT-BHQ1	(42)
GLAS	*infB*	CTinfF1: CGACTTACTTGAAGCCATTCTTCTT CTinfR1: CCGCTTGCCATACCCTCTT CTinfP: FAM-ATCGGAAGTATTAGAATTAAGTGC-TAMRA	(44)
MHP	*p102*	P102f: GTCAAAGTCAAAGTCAGCAAAC P102r: AGCTGTTCAAATGCTTGTCC P102 probe: Cy5-ACCAGTTTCCACTTCATCGCCTCA-BHQ2	(24)
MHR	*p37*	Mhr-p37-RT-F: TATCTCATTGACCTTGACTAAC Mhr-p37-RT-R: ATTTTCGCCAATAGCATTTG Mhr-p37-Probe: FAM-CATCCTCTTGCTTGACTACTCCTG-BHQ1	(43)
STREP	*recN*	SSrecN-F: CTACAAACAGCTCTCTTCT SSrecN-R: ACAACAGCCAATTCATGGCGTGATT	(13)
PCV2	ORF1	P1570: TGGCCCGCAGTATTCTGATT P1642: CAGCTGGGACAGCAGTTGAG P1591 (probe): FAM-CCAGCAATCAGACCCCGTTGGAATG-TAMRA	(29)
PCV3	*rep*	PCV3_353_F: TGACGGAGACGTCGGGAAAT PCV3_465_R: CGGTTTACCCAACCCCATCA PCV3_418_probe: FAM-GGGCGGGGTTTGCGTGATTT-BHQ1	(8)
swIAV	M gene	IAV-M1-F: AGATGAGTCTTCTAACCGAGGTCG IAV-M1-R1: TGCAAAAACATCTTCAAGTCTCTG IAV-M1-R2: TGCAAAGACACTTTCCAGTCTCTG IAV-M1-FAM (probe): FAM-TCAGGCCCCCTCAAAGCCGA-BHQ1	(16)

1 APP – *Actinobacillus pleuropneumoniae*; F – forward; R – reverse; GLAS – *Glaesserella parasuis*; MHP – *Mycoplasma hyopneumoniae*; MHR – *Mycoplasma hyorhinis*; STREP – *Streptococcus suis*; PCV2/3 – porcine circovirus 2/3; ORF1 – open reading frame 1; swIAV – swine influenza A virus

### Statistical analysis

Confidence intervals (CI) for seroprevalence were calculated using the Wilson score method. The normality of the CVPC scores was first assessed with the Shapiro–Wilk test. Depending on the outcome, either Student’s *t*-test for independent samples or the Mann–Whitney U test was used to compare mean CVPC scores depending on the presence or absence of specific pathogens or their combinations. The Kruskal–Wallis test was performed, followed by Dunn’s multiple comparisons test for mean CVPC scores of patterns of co-infection. Microsoft Excel 2019 (version 2409; Microsoft, Redmond, WA, USA), the Real Statistics Resource Pack for Excel (Release 9.1.1) (47) and GraphPad Prism (Version 9.0; GraphPad Software, Boston, MA, USA) were used for data analysis. Probability values of <0.05 were considered statistically significant.

## Results

### Farm level

Among the examined farms, there was not a single one where the presence of at least one of the tested pathogens was not detected. Most frequently the tested herds carried five or six pathogens, which were each in 40.9% (9/22) of the herds. In 9.1% (2/22) of herds, three pathogens were present simultaneously. In addition, four- and seven-pathogen co-occurrence was detected in one herd each 4.5% (1/22).

The occurrence of specific pathogens at the farm level (n = 22) was as follows: APP 4.5% (95% CI 0.8 –21.8), GLAS 95.5% (95% CI 78.2–99.2), MHP 95.5% (95% CI 78.2–99.2), MHR 68.2% (95% CI 47.3–83.6), STREP 9.1% (95% CI 2.5–27.8), PRRSV1 54.5% (95% CI 34.7–73.1), PRRSV2 9.1% (95% CI 2.5–27.8), PCV2 86.4% (95% CI 66.7–95.3), PCV3 95.5% (95% CI 78.2 –99.2) and swIAV 9.1% (95% CI 2.5–27.8). The within-herd prevalence of positive herds is presented in Table 2.

**Table 2.** Within-herd prevalence of respiratory pathogens in PCR-positive Polish pig herds

Pathogen	Number of positive farms (of 22 total)	Within-herd prevalence (%)	95% confidence interval	Range	Median
APP	1	20.0	N/A	N/A	N/A
GLAS	21	67.6	55.8–79.4	10.0–95.0	75.0
MHP	21	92.4	85.7–99.1	35.0–100	100
MHR	15	17.3	11.4–23.2	5.0–45.0	15.0
STREP	2	27.5	17.1–37.9	20.0–35.0	27.5
PRRSV1	12	40.0	23.0–57.0	5.0–100	30.0
PRRSV2	2	22.5	5.2–39.8	10.0–35.0	22.5
PCV2	19	51.3	36.6–66.0	5.0–100	45.0
PCV3	21	32.4	22.4–42.4	5.0–80.0	20.0
swIAV	2	20.0	13.1–26.9	15.0–25.0	20.0

2 APP – *Actinobacillus pleuropneumoniae*; GLAS – *Glaesserella parasuis*; MHP – *Mycoplasma hyopneumoniae*; MHR – *Mycoplasma hyorhinis*; STREP – *Streptococcus suis*; PRRSV1/2 – porcine reproductive and respiratory syndrome virus 1/2; PCV2/3 – porcine circovirus 2/3; swIAV – swine influenza A virus; N/A – not applicable

The most common combination of pathogens detected at the farm level was GLAS+MHP+MHR+PRRSV1+PCV2+PCV3, which was revealed in 31.8% (7/22) of herds. The second most popular combination was GLAS+MHP+MHR+PCV2+PCV3 and was detected in 13.6% (3/22) of the herd. Other combinations were found only in individual herds. The combinations of swine respiratory pathogens discovered at the farm level are shown in Table 3.

**Table 3.** Combinations of swine respiratory pathogens at the farm level in slaughtered Polish pigs examined by PCR

Combination of pathogens	Number of positive farms (of 22 total)	Prevalence (%)	95% Confidence interval
APP+GLAS+MHP+MHR+PRRSV1+PCV2+PCV3	1	4.5	0.8–21.8
GLAS+MHP+MHR+PRRSV1+PCV2+PCV3	7	31.8	16.4–52.6
GLAS+MHP+MHR+PRRSV1+PCV3+swIAV	1	4.5	0.8–21.8
GLAS+MHP+PRRSV1+PCV2+PCV3+swIAV	1	4.5	0.8–21.8
GLAS+MHP+MHR+PCV2+PCV3	3	13.6	4.7–33.3
GLAS+MHP+MHR+PRRSV2+PCV2	1	4.5	0.8–21.8
GLAS+MHP+PRRSV1+PCV2+PCV3	1	4.5	0.8–21.8
GLAS+MHP+PRRSV2+PCV2+PCV3	1	4.5	0.8–21.8
GLAS+MHP+STREP+PCV2+PCV3	1	4.5	0.8–21.8
GLAS+MHP+STREP+PRRSV1+PCV3	1	4.5	0.8–21.8
GLAS+MHP+MHR+PCV2+PCV3	1	4.5	0.8–21.8
GLAS+MHP+MHR+PCV2	1	4.5	0.8–21.8
MHP+PCV2+PCV3	1	4.5	0.8–21.8
GLAS+PCV2+PCV3	1	4.5	0.8–21.8

3 APP – *Actinobacillus pleuropneumoniae*; GLAS – *Glaesserella parasuis*; MHP – *Mycoplasma hyopneumoniae*; MHR – *Mycoplasma hyorhinis*; PRRSV1/2 – porcine reproductive and respiratory syndrome virus 1/2; PCV2/3 – porcine circovirus 2/3; swIAV – swine influenza A virus; STREP – *Streptococcus suis*

Cranioventral pulmonary consolidations were observed in slaughtered pigs in all herds. The mean CVPC score was 3.67 (standard deviation (SD) ± 2.01), ranging from 0.55 to 8.80, with a median value of 3.3. The mean CVPC scores were significantly higher in herds where MHR was detected – 4.41 (SD ± 2.00) in comparison with 2.09 in MHR-negative herds (SD ± 0.74; P-value = 0.0074). No such differences were observed for the other pathogens tested. The overall comparison showed no differences in CVPC scores for different pathogen combinations. However, when comparing the mean CVPC score for the most common combination (GLAS+MHP+MHR+PRRSV1+PCV2+PCV3) of 5.19 (SD ± 2.07) with the mean CVPC scores for all other combinations of 2.96 (SD ± 1.54), a statistically significant difference was found (P-value = 0.041). The mean CVPC score in herds where five pathogens or fewer were detected was 2.55 (SD ± 1.53), whereas in herds where six pathogens or more were detected, it was 4.27 (SD ± 1.96); the difference was statistically significant (P-value = 0.038).

### Animal level

Among the 440 pigs included in the study, only 2 (0.5%) animals yielded samples which were negative for all 10 pathogens. In most of the pig lungs (34.1%; 150/440), two infectious agents were detected, and the co-occurrence of three pathogens was observed in 30.9% (136/440) of pig lungs. The simultaneous presence of four pathogens in the lung samples was detected in 17.5% (77/440). A single pathogen was revealed in 11.8% (52/440) of lung samples, and only 5.2% (23/440) of the pig lungs tested were positive for five respiratory pathogens.

The occurrence of specific pathogens at the animal level (n = 440) was as follows: APP 0.9% (95% CI 0.4–2.3), GLAS 64.5% (95% CI 60.0–68.9), MHP 88.2% (95% CI 84.8–90.9), MHR 11.8% (95% CI 9.1–15.2), STREP 2.5% (95% CI 1.4–4.4), PRRSV1 21.8% (95% CI 18.2–25.9), PRRSV2 2.0% (95% CI 1.1–3.8), PCV2 44.3% (95% CI 39.8–49.0), PCV3 30.9% (95% CI 26.8–35.4) and swIAV 1.8% (95% CI 0.9–3.5).

At the animal level, 55 different patterns of co-infection were identified. The most common pattern of infections detected at the animal level was the simultaneous occurrence of GLAS and MHP, which was observed in 13.2% of pig lungs (58/440, 95% CI 10.3–16.7). *Mycoplasma hyopneumoniae* alone was detected in 8.6% of samples (38/440, 95% CI 6.4–11.6) as the next most common pattern. Other pathogen infections observed frequently at the animal level were GLAS+MHP+PCV2, present in 8.0% (95% CI 5.8 –10.9); GLAS+MHP+PCV2+PCV3, noted in 6.8% (95% CI 4.8–9.6); GLAS+MHP+PRRSV1, constituting 6.1% (95% CI 4.3–8.8); and MHP+PCV2, for which the percentage was 5.9% (95% CI 4.1–8.5). All of the patterns of co-infection are presented in Table 4.

**Table 4.** Mean cranioventral pulmonary consolidation (CVPC) scores for different patterns of co-infection detected in the lungs of slaughtered Polish pigs

Pattern of co-infection	Number of pigs affected (of 440 total)	Mean CVPC score (± SD)	P-value*
GLAS+MHP+MHR+PCV2	5	9.00	0.0517
MHP+MHR+PCV3	1	9.00	0.2065
MHP+MHR+PCV2	1	8.00	0.2519
GLAS+MHP+MHR+PRRSV2	1	8.00	0.2519
GLAS+MHP+MHR+PRRSV1+PCV2	4	7.25	0.4248
GLAS+MHP+PRRSV2	1	7.00	0.3020
GLAS+MHP+PRRSV1+PCV3	13	6.46	**0.0321**
GLAS+MHP+PRRSV1+PCV2+PCV3	5	6.20	0.5488
GLAS+MHP+PRRSV1+PCV2	13	6.08	0.3212
APP+MHP+PRRSV1	2	6.00	0.2924
MHP+PRRSV1+PCV2	1	6.00	0.3729
GLAS+STREP+PCV2+PCV3	1	6.00	0.3729
GLAS+MHP+MHR+PCV3	4	5.75	0.1654
MHP+PRRSV1+PCV2+PCV3	2	5.50	0.9606
MHP+MHR	2	5.00	0.8483
GLAS+MHP+PRRSV2+PCV2	1	5.00	0.4993
MHP+PRRSV1+PCV3+swIAV	1	5.00	0.4993
GLAS+MHP+MHR	20	4.95	0.5113
MHP+PRRSV1	13	4.85	0.5047
GLAS+MHP+MHR+PCV2+PCV3	6	4.67	0.7993
MHP+PRRSV2	2	4.50	0.4687
GLAS+MHP+MHR+PRRSV1	2	4.50	0.7305
GLAS+MHP+MHR+PRRSV1+PCV3	2	4.50	0.8652
MHP	38	4.16	reference
PCV3	1	4.00	0.7172
MHP+PRRSV1+PCV3	3	4.00	0.8763
MHP+MHR+PCV2+PCV3	2	4.00	0.6539
APP+GLAS+MHP+PRRSV1+PCV2	1	4.00	0.7172
GLAS+MHP+PRRSV1+PCV3+swIAV	4	4.00	0.9670
MHP+PCV3	19	3.95	0.9260
MHP+STREP	3	3.67	0.8244
GLAS+MHP+PRRSV1	27	3.48	0.4428
MHP+PCV2+PCV3	17	3.47	0.7309
GLAS+MHP+PCV2	35	3.37	0.3623
GLAS+MHP+PCV3	9	3.33	0.5240
GLAS+MHP	58	3.17	0.2953
GLAS+MHP+PCV2+PCV3	30	3.07	0.2003
MHP+PCV2	26	3.04	0.3147
MHP+MHR+PRRSV1	1	3.00	0.9734
MHP+PRRSV2+PCV3	4	3.00	0.9067
GLAS	4	2.00	0.2558
GLAS+MHP+swIAV	1	2.00	0.5901
GLAS+MHP+STREP+PCV2	2	2.00	0.4519
APP+MHP+PRRSV1+PCV2+PCV3	1	2.00	0.5901
GLAS+MHP+STREP	4	1.75	0.1960
GLAS+PCV2+PCV3	7	1.14	**0.0199**
GLAS+PCV2	21	1.05	**0.0002**
PCV2+PCV3	4	1.00	0.0526
MHP+PCV2+swIAV	1	1.00	0.3226
PCV2	8	0.38	**0.0006**
None	2	0.50	0.0807
swIAV	1	0.00	0.1320
GLAS+MHR	1	0.00	0.1320
GLAS+STREP	1	0.00	0.1320
GLAS+PRRSV1+PCV2	1	0.00	0.1320

* – pairwise comparisons of MHP (reference) with other patterns of infection or co-infection; SD – standard deviation; GLAS – *Glaesserella parasuis*; MHP – *Mycoplasma hyopneumoniae*; MHR – *Mycoplasma hyorhinis*; PCV2/3 – porcine circovirus 2/3; PRRSV1/2 – porcine reproductive and respiratory syndrome virus 1/2; APP – *Actinobacillus pleuropneumoniae*; STREP – *Streptococcus suis*; swIAV – swine influenza A virus; P-values in bold are statistically significant

Cranioventral pulmonary consolidations were observed in 79.3% (95% CI 75.3–82.8) of slaughtered pigs. The frequency of CVPC lesions was significantly higher (P-value < 0.001) in animals where MHP was detected, at 83.2% compared with 50% in pigs with lungs negative for MHP. No statistically significant correlations were found between the occurrence of the other pathogens investigated in lung tissue and the presence of CVPC lesions.

**Fig. 1.**
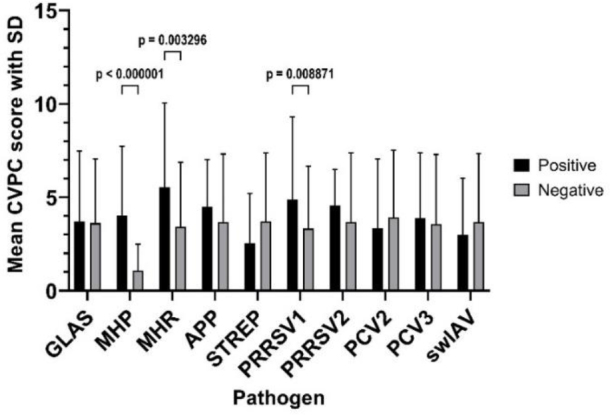
The mean values of cranioventral pulmonary consolidation (CVPC) lesion scores in the lungs of slaughtered Polish pigs depending on the infectious agent. SD – standard deviation; GLAS – *Glaesserella parasuis*; MHP – *Mycoplasma hyopneumoniae*; MHR – *Mycoplasma hyorhinis*; APP – *Actinobacillus pleuropneumoniae*; STREP – *Streptococcus suis*; PRRSV1/2 – porcine reproductive and respiratory syndrome virus 1/2; PCV2/3 – porcine circovirus 2/3; swIAV – swine influenza A virus

The mean CVPC score was 3.67 (SD ± 3.65), ranging from 0 to 21, with a median value of 3. The mean CVPC score was significantly higher in animals infected by MHP, being 4.02 (SD ± 3.71) in comparison with 1.08 in MHP-negative pigs (SD ± 1.40; P-value < 0.001). Other significant differences between mean CVPC scores with pathogen detection and these scores without it were observed for MHR (5.54 (SD ± 4.47) *vs* 3.42 (SD ± 3.44); P-value < 0.003) and PRRSV1 (4.88 (SD ± 4.38) *vs* 3.33 (SD ± 3.33); P-value < 0.009). The mean CVPC scores depending on the infectious agent are presented in Fig. 1. Comparisons of mean CVPC scores of lungs positive only for MHP with the scores of lungs with co-infections revealed a significantly different and higher score in one case. In pigs infected by GLAS+MHP+PRRSV1+PCV3, the mean CVPC value was 6.46 (SD ± 3.75) and was higher than the 4.16 (SD ± 3.87) in MHP-only-positive pigs. In pigs positive for PCV2 only, for GLAS+PCV2 and for GLAS+PCV2+PCV3, the mean values of CVPC were lower than those in pigs positive only for MHP and amounted to 0.38 (SD ± 0.99), 1.05 (SD ± 1.05) and 1.14 (SD ± 1.12), respectively. The results for all patterns of infection and co-infection are shown in Table 4.

The mean CVPC score in pigs where two pathogens or fewer were detected in lungs was 3.09 (SD ± 3.23), whereas in herds where three pathogens or more were detected, it was 4.11 (SD ± 3.83); the difference was statistically significant (P-value = 0.004).

## Discussion

Cranioventral pulmonary consolidations are among the most commonly observed lung lesions in pigs post slaughter (22). Their occurrence is associated with significant economic losses in pig farms (23). Moreover, CVPC lesions alone cause about 50% of lung rejections after slaughter, significantly impacting processing efficiency (33).

The results of this study confirm that CVPC lesions are highly prevalent on commercial pig farms. In our study, such lesions were observed in pigs from all investigated herds with a prevalence of 79.3%, and their prevalence corresponded well with that of 85.16% in lungs previously observed by Przyborowska *et al*. (33) in Wielkopolskie voivodeship. However, on a national scale, the occurrence of such changes was lower and ranged between 24 and 58% (33, 34). The difference may be a consequence of the higher density of pig production in the Wielkopolskie voivodeship. It was reported that the greater the concentration of production, the greater the risk for respiratory disease in pigs (38). The CVPC results may also be influenced by the sample size, *i.e*., the number of herds from which the pigs were sourced, as well as by the cross-sectional rather than longitudinal nature of the study, which may be relevant given the seasonal occurrence of lung lesions (45). The prevalence of CVPC lesions varied significantly across different countries worldwide. The lowest percentage of affected pigs was reported in New Zealand (27), while the highest was observed in Brazil (9). There are European countries where CVPC lesions are only sporadically noted. These are the countries where MHP has been practically eradicated (21). In other European countries, the percentage of lungs with CVPC lesions ranged from 24% in Belgium and 27% in the UK to 72% in France and 73% in Germany (4, 17, 19, 25).

Naturally, CVPC is associated with MHP infections in pigs and these lung changes are sometimes referred to as *Mycoplasma*-like lesions or enzootic pneumonia-like lesions (10, 30). On the other hand, it has been demonstrated that such lung lesions are not pathognomonic for MHP infection (22). Similar changes are also observed in the course of other bacterial and viral diseases, such as infections with swIAV (32). The possibility that such lesions may be caused by bacterial pathogens other than MHP is further supported by studies showing that 59% of lungs from slaughtered pigs originating from MHP-free farms exhibited mild CVPC lesions affecting less than 10% of lung tissue (19). The development of mild CVPC lesions may also be related to the action of non-infectious factors such as inhalation of ammonia or dust (11).

Whether at the herd level or the individual animal level, MHP was the most commonly occurring pathogen. Similar results were obtained in previous studies conducted in France, Germany and Belgium (5, 19, 26). As noted by Maes *et al*. (22), the prevalence of CVPC lesions has remained unchanged over the years despite advancements in control methods, including new vaccines and improved biosecurity measures. The present investigation’s high prevalence of MHP observed in lung samples from fattening pigs, despite the widespread implementation of immunoprophylactic programmes targeting this pathogen in the studied herds, suggested that vaccination had not eliminated the pathogen nor prevented its transmission between animals. This conclusion is supported by previous studies which found that vaccination against MHP reduced but did not completely eliminate pathogen circulation and transmission at the farm level (21). Vaccination was shown to alleviate clinical symptoms, decrease the severity of lung lesions and reduce macrophage infiltration in the bronchus-associated lymphoid tissue region (46).

The high percentage of lungs in which MHP was detected in our study closely corresponds with the percentage of lungs showing CVPC lesions. We found that the presence of MHP in the lungs of the examined pigs was associated with a higher frequency of CVPC lesions and a higher lesion score. Our findings aligned with previous studies that clearly suggested a link between the presence of MHP in pig lungs and the occurrence of anatomopathological lesions (7, 31). However, respiratory disease in pigs was often polymicrobial and multifactorial in nature (28). The presence of multiple pathogens or co-infections could prolong disease duration and exacerbate its severity (37). The interactions between respiratory pathogens in swine infections were described as highly complex, with mechanisms of action that may be additive or synergistic (41). For instance, prior infection with swIVA could have promoted MHP colonisation in the respiratory tract (31). Additionally, co-infection with MHP and PRRSV could have aggravated clinical severity by compromising immune responses and facilitating secondary bacterial infections, such as those caused by APP or *Pasteurella multocida* (35).

In our study, the simultaneous presence of multiple pathogens was frequently detected at both the herd and individual animal levels. Notably, only about 12% of lung samples contained a single pathogen, reinforcing the widely accepted notion that co-infections are a key factor contributing to lung lesions in pigs. These findings are consistent with previous research (2, 35). Our results demonstrated that the presence of multiple pathogenic microorganisms at both the herd and individual levels correlated with increased severity of lung lesions. The observed correlation between pathogen detection and CVPC supported the findings of other studies, which demonstrated that increased pathogen load was often associated with more extensive lung damage (44). Studies in Belgium and Italy similarly demonstrated that pigs harbouring three or more respiratory pathogens exhibited significantly more severe lesions than those infected with only one or two agents (21, 25).

Additionally, we confirmed that PRRSV1 and MHR played a significant role in the development of CVPC lesions in pigs. Porcine reproductive and respiratory virus 1, in particular, facilitated secondary bacterial infections, such as those caused by *Pasteurella multocida* and STREP (35). Although CVPC lesions have been sporadically reported as a consequence of PRRSV1 infection, they are not considered a characteristic feature of this virus (36). The precise role of MHR in CVPC development remains unclear. In several studies it was found that lung lesions typical for MHP infections could also be provoked by infection with MHR without involvement of MHP (1). Contrastingly, Luehrs *et al*. (19) suggested that MHR alone did not induce lung lesions resembling those observed in MHP infections in fattening pigs. Other studies also indicated that MHR did not act as a primary causative agent but instead exacerbated disease progression initiated by MHP (6). In our study, MHR was detected exclusively in co-infections with other pathogens. In the vast majority of cases, this included co-infection with MHP. Only in one case, in a lung where no lesions were found, was MHR detected together with GLAS without the involvement of MHP.

Our findings also revealed that co-infection with GLAS, MHP, PRRSV1 and PCV3 was associated with more severe lesions in the lungs than infections with MHP alone. This is consistent with previous reports highlighting the role of bacterial-viral co-infections, particularly those involving MHP and PRRSV, in the progression of respiratory disease in pigs (37). Furthermore, PCV3, a relatively recently discovered virus, was implicated in the exacerbation of PRDC (14).

Importantly, our study found that the mere presence of circovirus in lung tissue was insufficient to induce CVPC lesions. Pigs infected solely with PCV2 or coinfected with PCV2, PCV3 and/or GLAS exhibited less pronounced lung lesions than those infected with MHP alone. This aligns with recent insights into PCV2 infections, which demoted "PCV2 lung disease" from being a distinct diagnostic entity and considered it a component of PCV2 systemic disease or PRDC (39). Additionally, PCV2 was recognised as a contributing factor in PRDC, particularly in the context of co-infections with PRRSV (3). However, experimental coinoculation of PCV2 and MHP did not lead to a synergistic exacerbation of the clinical symptoms or lung lesions associated with either pathogen (40).

In this study, we focused on the relationship between the occurrence of CVPC lesions and the presence of selected respiratory pathogens in the lungs of slaughtered pigs. We acknowledge that our chosen method, based on detecting the genetic material of pathogenic microorganisms in lung samples, may not provide a complete picture. The lungs were previously considered sterile; however, current research indicated that their microbiota closely resembled the oral microbiota (18). *Sphingobium* was among the genera in heaviest presence in both healthy and lesioned lungs, but *Methylotenera, Prevotella* and *Lactobacillus* were the other predominant genera in the microbiota of healthy lungs, whereas *Mycoplasma, Ureaplasma, Haemophilus* and *Phyllobacterium* joined *Sphingobium* as the predominant microbial communities in lungs exhibiting severe lesions (12). A limitation of this study is its reliance on PCR detection of pathogen DNA/RNA, which does not distinguish between active infection and residual genetic material. However, detecting antibodies instead and linking their presence to lung lesions in pigs would have been much more challenging because of the potential detection of vaccine-induced antibodies or antibodies from past infections that no longer result in visible lung lesions. Future research should incorporate histopathological analysis and bacterial culture to confirm infection status.

## Conclusion

This study reinforced the complexity of respiratory infections in pigs, emphasising the need for comprehensive disease control strategies that address both primary and secondary pathogens. At the same time, these findings confirm the primary role of MHP in the development of CVPC lesions in pigs. Further investigations into co-infection dynamics and intervention efficacy will be essential for improving swine health and productivity.
